# An Unusual Case of a Vulva Tumor in an Adolescent: A Case Report and Literature Review

**DOI:** 10.15388/Amed.2026.33.1.19

**Published:** 2026-07-22

**Authors:** Hayriye Nihan Karaman Ayyildiz, Zekeriya İlçe, İlkay Tosun, Funda Tekkesin

**Affiliations:** 1 Health Sciences University Ümraniye Training and Research Hospital Pediatric Surgery Clinic, Türkiye; 2 Department of Pediatric Surgery, Umraniye Training and Research Hospital, Umraniye Istanbul, Türkiye; 3 Department of Pathology, Umraniye Training and Research Hospital, Umraniye Istanbul, Türkiye; 4 Department of Pediatric Hematology-Oncology, Umraniye Training and Research Hospital, Umraniye Istanbul, Türkiye

**Keywords:** adolescent, vulva, primitive neuroectodermal tumors, extraosseous Ewing’s sarcoma, paauglė, vulva, primityvūs neuroektoderminiai navikai, ekstraosinė Ewingo sarkoma

## Abstract

**Background:**

We present the case of a 15-year-old adolescent with swelling in the left vulva that was found to have a tumor measuring approximately 20x15 cm in her vulva. Ewing’s sarcoma/primitive neuroectodermal tumors (ES/PNET) are rare and malignant.

**Materials and Methods:**

Preoperative magnetic resonance imaging and computed tomography performed to screen for metastasis were negative. The mass was surgically excised in total.

**Results:**

Immunohistochemistry showed the mass to be ES/PNET. The patient underwent positron emission tomography-computed tomography which revealed metastasis to inguinal lymph nodes and bone. While under chemotherapy for stage-4 ES/PNET, the patient was found to have lung metastasis. She died due to recurrent chemotherapy complications during treatment.

**Conclusions:**

This rare case highlights that significant of early diagnosis and multidisciplinary approach to treatment of ES/PNET cases.

## Abstract (it)

**Įvadas:**

Pateikiame 15-metės paauglės, kuriai buvo nustatytas kairės vulvos patinimas, o vėliau – maždaug 20x15 cm dydžio navikas vulvoje, atvejį. Ewingo sarkoma ir primityvieji neuroektoderminiai navikai (ES/PNET) yra reti ir piktybiniai.

**Medžiaga ir metodai:**

Priešoperacinė magnetinio rezonanso tomografija ir kompiuterinė tomografija, atliktos siekiant nustatyti metastazes, buvo neigiamos. Navikas visiškai pašalintas chirurginiu būdu.

**Rezultatai:**

Imunohistocheminis tyrimas parodė, kad navikas yra ES/PNET. Pacientei buvo atlikta pozitronų emisijos tomografija ir kompiuterinė tomografija, kuriomis buvo nustatytos metastazės į kirkšnies limfmazgius ir kaulus. Vykstant ketvirtos stadijos ES/PNET chemoterapijai, pacientei buvo nustatytos plaučių metastazės. Ji mirė dėl pasikartojančių chemoterapijos komplikacijų gydymo metu.

**Išvados:**

Šis retas atvejis pabrėžia ankstyvos diagnozės ir daugiadisciplininio požiūrio į ES/PNET atvejų gydymą svarbą.

## Introduction

Ewing’s sarcoma/primitive neuroectodermal tumors (ES/PNETs) are malignant neoplasms of the skeletal system and are seen mostly in the bones. Primary extraosseous ES/PNETs are rare and account for 1% of soft tissue sarcomas [1]. They most commonly involve the chest wall, lower extremities, and paravertebral region, and less commonly involve the pelvis and gluteal region, retroperitoneum, and upper extremities [2].

ES/PNET of the female genital tract is uncommon and usually involves the vulva, vagina, uterus, and ovaries [3,4,5]. Reported cases of vulvar ES/PNET are extremely rare [6]. ES/PNET of the vulva mostly occurs in the second decade of life and is rare in children. Our review of the literature revealed 10 case reports involving children [4,7–15]. Since some of these patients died during treatment, knowledge on this group is limited. Information on ES/PNET of the vulva and its treatment is primarily based on data from adults. Against this background, we aimed to contribute to the literature by evaluating the information and data about the patient who applied to our clinic in light of the literature available.

## Materials and Methods

**Fig. 1:**
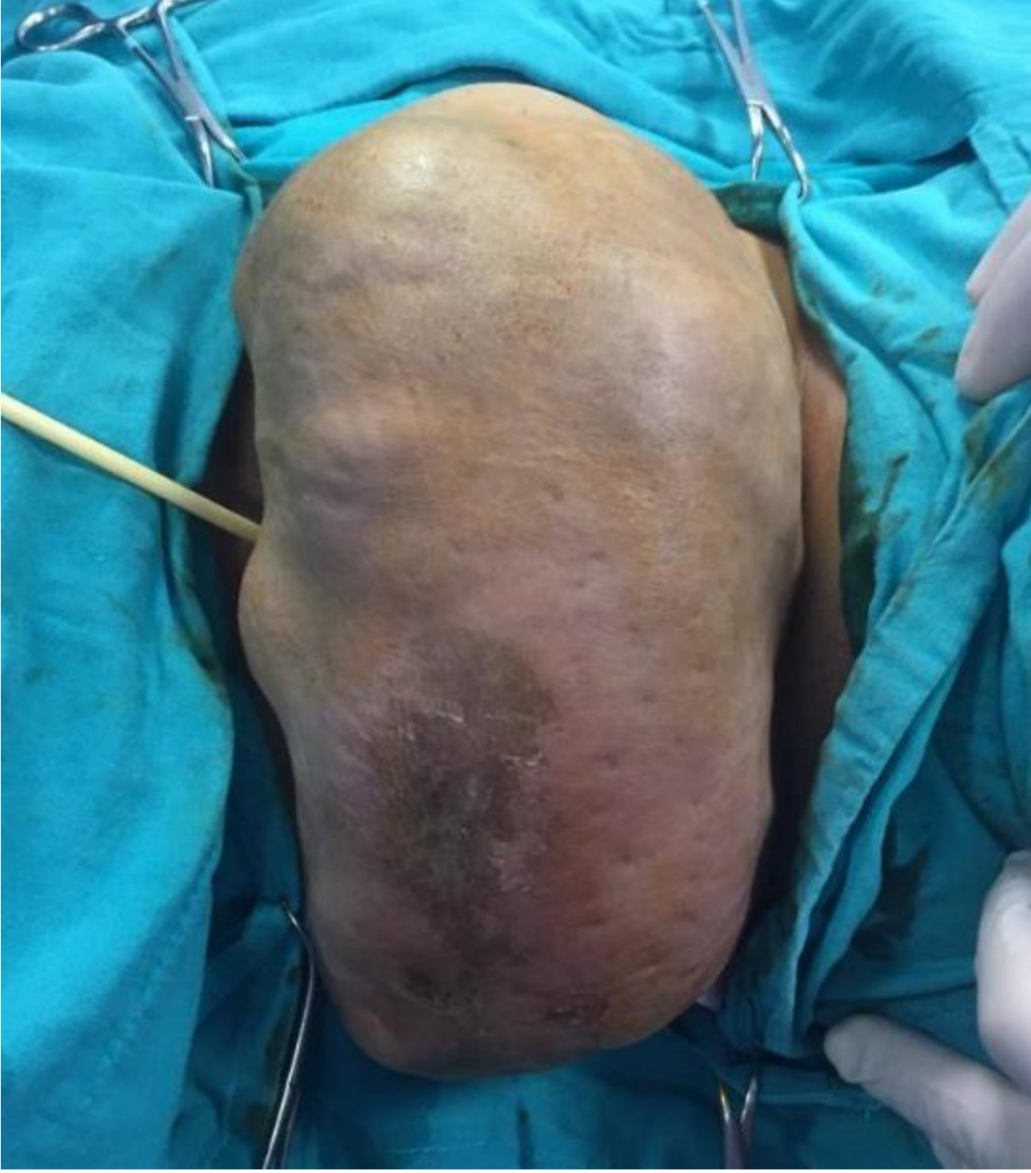
Fifteen-year-old girl presented with a giant vulval mass

A 15-year-old female patient presented with left vulvar swelling. The patient’s history indicated that the mass had been present for a year. However, the patient did not inform her family about it. It was only when the swelling grew rapidly over the two previous months, that she ended up consulting her family; this is how the patient came to see the physician. The patient had normal physical development for her age, and her family history was unremarkable. Physical examination revealed a mass of 20x15x15 cm in the left vulva that was firm, lobulated, non-tender, involving the left mons pubis, extending to the left inguinal region, with ulcerations on it (Fig. 1); there was no lymphadenopathy.

**Fig. 2:**
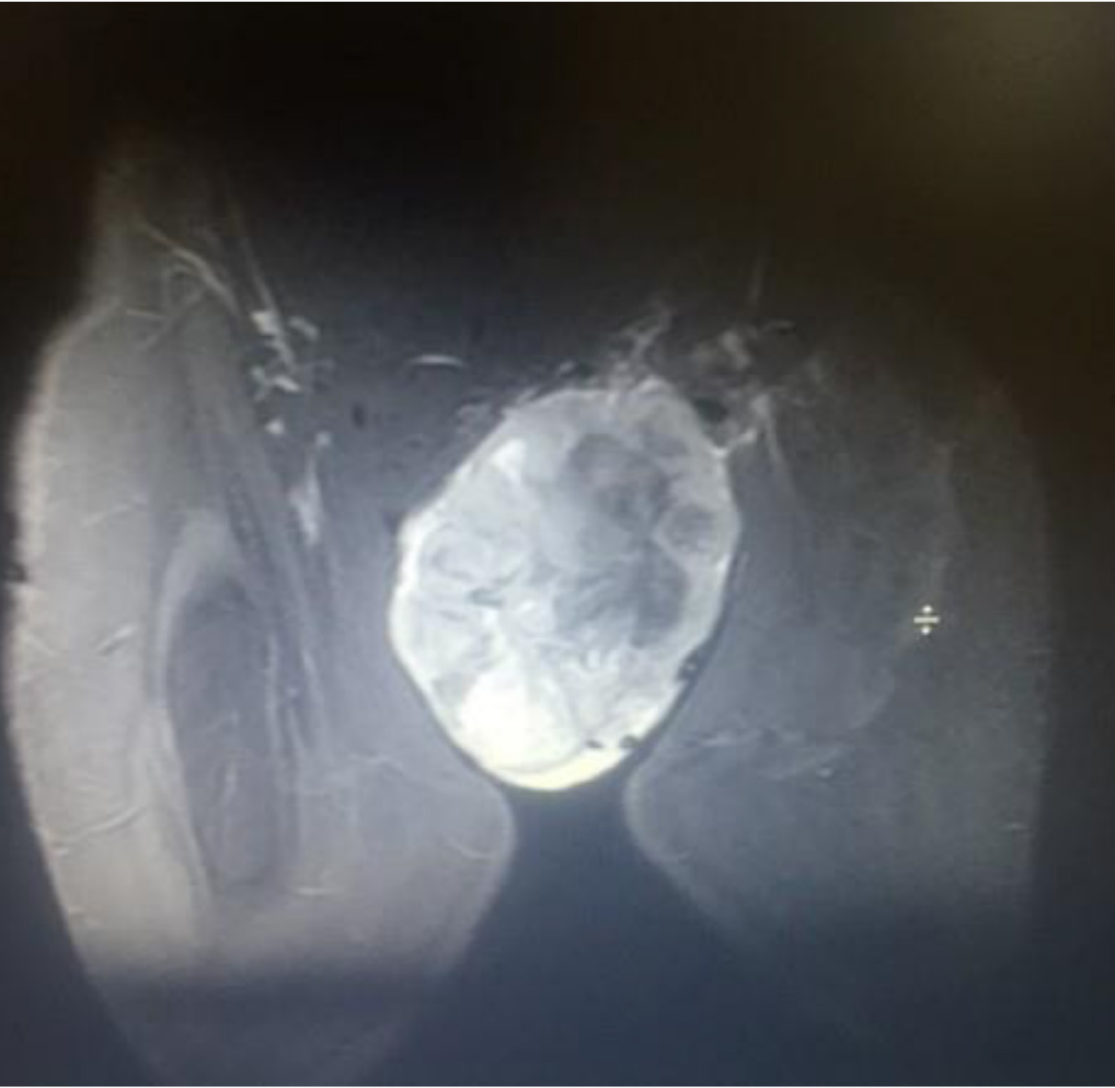
MR image of the mass in the vulva

Liver and kidney function test results, as well as tumor markers (CA125, CA19-9, CEA), were within normal limits. Pelvic magnetic resonance imaging (MRI) found a macrolobulated giant mass lesion measuring 21x14.5x13 cm at the level of the vulva; it was more prominent on the left side, filling the midline to a great extent, showing significant extrinsic extension in the caudal direction, largely limited by subcutaneous fatty tissue planes (Fig. 2).

**Fig. 3:**
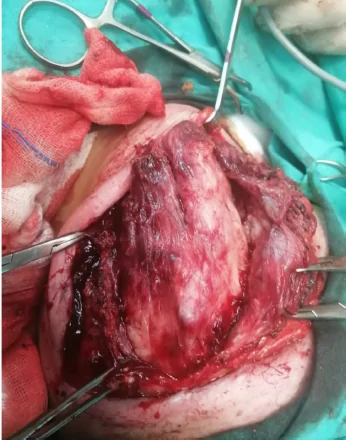
Surgical image of the mass in the vulva

The patient was evaluated by the pediatric oncology council, which developed a treatment plan. Preoperative computed tomography (CT) of the abdomen and thorax revealed no pathology. Surgical resection was performed in line with the planned total excision. During the surgical procedure, the mass was found to be an irregular, hard tissue mass of 20x15x15 cm, adherent to the vulva, progressing towards the vagina but clearly separated without invasion into the vagina. An incision was performed over the vulvar mass to open the skin and the subcutaneous tissue. The mass was dissected off the surrounding tissues and the base. Surgical hemostasis was achieved. The mass was completely excised, leaving the surrounding tissue intact. The procedure was concluded with vulvoplasty, involving the suturing of vulvar layers (Fig. 3).

**Fig. 4:**
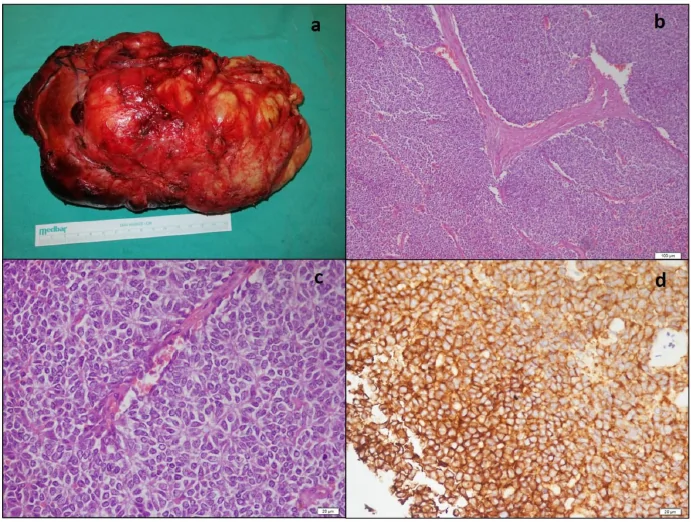
**a**. Pathological macroscopic image, **b**. Pathological – tumor composed of monomorphic small cells with fibrous bands, **c**. Tumor cells with small, narrow cytoplasm, salt-pepper chromatin forming pathological rosette structures, **d**. Pathological – diffuse membranous CD99 expression in tumor cells

Pathological examination revealed the mass to be firm and measuring 21x3x9 cm in size (Fig. 4a). Microscopic examination showed a necrotic tumor with fibrous bands (Fig. 4b), diffuse infiltration, and sporadic rosette-like structures (Fig. 4c). The tumor cells were small, monomorphic and had narrow cytoplasm and thin chromatin. Immunohistochemistry showed diffuse membranous staining with CD99 (Fig. 4d) and positive nuclear staining with FLI-1. Molecular testing was planned to confirm the diagnosis. RT-PCR was performed to investigate the EWSR-FLI1 fusion gene and detected EWSR-FLI1 T (11; 22) (q24; q12) type 1 fusion. Histomorphologic, immunohistochemical, and molecular test results led to a diagnosis of ES/PNET.

Postoperative PET/CT revealed hypermetabolic uptake in the left inguinal lymph nodes and right scapula at the level of the glenoid. Bilateral bone marrow aspiration and biopsy revealed no involvement. Based on a diagnosis of stage-4 ES/PNET, the patient was postoperatively started on chemotherapy in line with the Euro-Ewing-99 protocol. After six cycles of chemotherapy consisting of vincristine (1.5 mg/m^2^, day 1), ifosfamide (3000 mg/m^2^/day, days 1-2-3), Doxorubicin (20 mg/m^2^/day, days 1-2-3), and etoposide (150 mg/m^2^/day, days 1-2-3), the patient was enrolled in the autologous transplantation arm because her mass was > 200 ml and metastatic at diagnosis. After two cycles of chemotherapy, the patient’s autologous stem cells were collected and stored. At month 10 of the treatment, the patient developed lung metastasis. The patient was started on chemotherapy again, to which, radiotherapy was added. The patient was re-hospitalized due to fever and neutropenia and died at month 14 of the treatment due to chemotherapy-related complications.

The patient and her legal guardian gave verbal and written consent for the publication of this case report.

## Discussion

ES/PNET is considered a member of the Ewing’s family of tumors with similar prognosis and immunohistochemical features [1]. Extraosseous ES/PNET of soft tissues is uncommon and rarely involves the female genital tract. In adults, it usually involves the uterus and the vagina and rarely involves the vulva [3]. Review of literature showed a total of 27 cases of vulvar PNET in adults. While we were unable to find any cases of uterine and vaginal PNETs in children, we found a total of 10 vulvar PNETs in this group [4,7–15]. Vulvar PNET affecting children was first reported by Scherr et al. in a 10-year-old girl [7].

Vulvar ES/PNET should be differentiated from benign lesions involving the vulva and vagina, including epidermal inclusion cysts, Gartner duct cysts, and Bartholin cysts [4]. The definitive diagnosis of a mass in the vulva requires surgical excision and pathological examination. Imaging exams such as CT and PET/CT are required to evaluate metastases and plan treatment.

The differential diagnosis of ES/PNET should include other small round blue cell tumors such as rhabdomyosarcoma, neuroblastoma, lymphoma, small cell carcinoma, melanoma, cutaneous adnexal tumors, and Merkel cell carcinoma [9]. Histopathology, as well as immunohistochemical and cytogenetic analysis, helps differentiate between these pathologies. Markers CD99, a cell membrane glycoprotein, and FLI-1, a DNA-binding transcription factor, are positive in most cases of ES/PNET [16]. Negative desmin and MyoD1 rule out rhabdomyosarcoma, while negative epithelial and neuroendocrine markers rule out small cell carcinoma and Merkel cell carcinoma. Absence of LCA positivity rules out lymphoma. T (11; 22) (q24; q12) chromosomal translocation (EWS-FLI1 gene rearrangement) occurs in more than 90% of tumors and is highly specific for ES/PNET. Our patient was found to have diffuse membranous staining with CD99 (Fig. 4d) and positive nuclear staining with FLI-1 and was diagnosed with ES/PNET.

There are no standard treatment guidelines available for these rare tumors. Treatment of ES/PNET, mostly based on adult data, requires a multidisciplinary approach and includes surgical resection and adjuvant chemotherapy [3]. The role of radiotherapy in treatment is unknown. Surgical treatment consists of tumor biopsy, wide local excision of the tumor, and radical surgery [7,17]. The main chemotherapeutic agents used in the treatment of ES/PNET and extraosseous Ewing’s sarcomas include vincristine, doxorubicin, cyclophosphamide, actinomycin-D, ifosfamide, and etoposide [4].

Pelvic ES/PNET usually has more aggressive behavior and a worse prognosis than Ewing’s sarcoma of bone. Pelvic ES/PNET has been associated with a poor prognosis and low survival rate, especially in metastatic cases [4,7,17,18]. However, there are reports of relatively better outcomes for ES/PNET arising in the skin and other superficial sites. Therefore, prognosis depends on the tumor size, tumor location, early diagnosis, presence or absence of metastasis, and complete surgical removal of the tumor [9,19].

According to our review of the literature, the 10 reported cases of pediatric patients had tumor sizes ranging from 0.6 cm to 20 cm. All of the reported cases were histopathologically diagnosed as ES/PNET, and five of them developed metastases (50%). Four of these patients had pulmonary metastasis, and one had metastasis to inguinal and femoral lymph nodes [7,10–13]. A 17-year-old patient with pulmonary metastasis also had pelvic bone metastasis [12]. No distant or near metastasis was reported for the other five patients [4,8,9,14,15]. Of the 10 patients, four (40%) died between months 3 and 14 of treatment. One of these patients had pulmonary metastasis, one had pulmonary metastasis along with bone metastasis, and the other two patients had no metastasis [10,12,14,15]. Clinicopathologic characteristics of the patients are summarized in Table 1.

**Table 1:** Summary of Cases of Pediatric Vulvar Ewing’s Sarcoma in the Literature

Reference	Age	Location	Size (cm)	Pathology Evaluation	Metastasis	Treatment	Follow-up
Scherr et al. [7], 1994	10	Left Labia majora	6.5	HBA-71 positive	Inguinal and femoral Lymph Node	Surgery	N/A
Lazure et al. [8], 2001	15	Labia majora	20	CD99 positive	None	Surgery Chemotherapy	NED at 7 months
Fong et al. [9], 2008	17	Left Labia majora	2.1	CD99 positive FLI-1 positive RT-PCR positive	None	Surgery Chemotherapy	NED at 48 months
Halil et al. [10], 2011	14	Left Vulva	4.5	CD99 positive	Pulmonary	Surgery Chemotherapy Radiotherapy	DOD at 9 months
Kelling et al. [11], 2012	18	Labia majora	1.7	CD99 positive RT-PCR positive	Pulmonary	Surgery Chemotherapy Radiotherapy	NED at 3 months
Tunitsky et al. [4], 2014	15	Left Labia minora	5	CD99 positive RT-PCR positive	None	Surgery Chemotherapy	NED at 10 months
Narayanan et al. [12], 2014	17	Clitoris	3	MIC2 positive	Bone Pulmonary	Surgery Chemotherapy Radiotherapy	DOD 6 at months
Rekhi et al. [13], 2015	10	Left Vulva	20	CD99 positive FLI-1 positive	Pulmonary	Surgery Chemotherapy	NED at 18 months
Kakoti et al. [14], 2017	16	Left Vulva	15	CD99 positive FLI-1 positive	None	Chemotherapy	DOD
Xu et al. [15], 2019	3	Vulva	5	CD99 positive	None	Surgery Chemotherapy	DOD at 14 months
Ayyıldız et al., 2021 (Present case)	15	Left Vulva	20	CD99 positive FLI-1 positive	Inguinal Lymph Node Bone	Surgery Chemotherapy Radiotherapy	DOD at 14 months

*Note*. The following abbreviations were used: N/A: Not Available; NED: No evidence of disease; DOD: Dead of disease; RT-PCR: Reverse transcription-polymerase chain reaction.

Based on the above-mentioned reports and the delayed presentation of our patient, we performed CT and MRI scans, which revealed no metastasis. The mass was removed in total. Although the mass was completely removed and no metastasis was detected on MRI and CT, the postoperative oncology council decided to perform a PET/CT scan to guide treatment and avoid missed metastasis. This PET/CT showed involvement of the inguinal lymph nodes and bone. While under chemotherapy for stage-4 ES/PNET, the patient was intermittently hospitalized for neutropenia and fever, and developed pulmonary metastasis at month 10 of the treatment. While chemotherapy and radiotherapy were underway, the patient expired at month 14 of the treatment due to chemotherapy-related complications.

## Conclusion

Extraosseous ES/PNET in children is extremely rare, and all the available information is limited to case reports. Given the rare nature of the disease and relatively short follow-up periods, it is difficult to comment on long-term survival and relapse rates. It is essential to use biopsy to confirm the diagnosis and attempt total resection regardless of the size of the mass. Since metastasis will affect the treatment protocol, scanning for metastasis should be performed before chemotherapy. It should be kept in mind that CT and MRI may fail in scanning for metastasis, and PET/CT exams should be used when necessary. Detection of metastases and the size of the mass should not lead to a change in the surgical goal, and biopsy should be followed by total removal whenever possible.

## References

[1] Gostout BS, Lindor NM, DiMarco CS, Peethambaram PP, Clayton AC (2003). Pelvic primitive neuroectodermal tumor associated with a cluster of small round cell tumors: case report and review of current literature. *Gynecol Oncol*.

[2] Peres E, Mattoo TK, Poulik J, Warrier I (2005). Primitive neuroectodermal tumor (PNET) of the uterus in a renal allograft patient: a case report. *Pediatr Blood Cancer*.

[3] Xiao C, Zhao J, Guo P (2014). Clinical analysis of primary primitive neuroectodermal tumors in the female genital tract. *Int J Gynecol Cancer*.

[4] Tunitsky-Bitton E, Uy-Kroh MJS, Michener C, Tarr ME (2015). Primary Ewing sarcoma presenting as a vulvar mass in an adolescent: case report and review of literature. *J Pediatr Adolesc Gynecol*.

[5] Yang J, Guo Q, Yang Y, Zhang J, Lang J, Shi H (2012). Primary vulvar Ewing sarcoma/primitive neuroectodermal tumor: a report of one case and review of the literature. *J Pediatr Adolesc Gynecol*.

[6] Blattner JM, Gable P, Quigley MM, McHale MT (2007). Primitive neuroectodermal tumor of the uterus. *Gynecol Oncol*.

[7] Scherr GR, d’Ablaing G, Ouzounian JG (1994). Peripheral primitive neuroectodermal tumor of the vulva. *Gynecol Oncol*.

[8] Lazure T, Alsamad IA, Meuric S, Orbach D, Fabre M (2001). Sarcomes d’Ewing/tumeurs neuroectodermiques primitives périphériques de l’utérus et de la vulve chez l’enfant: deux localisations exceptionnelles [Primary uterine and vulvar Ewing’s sarcoma/peripheral neuroectodermal tumors in children: two unusual locations]. *Ann Pathol*.

[9] Fong YE, Lopez-Terrada D, Zhai QJ (2008). Primary Ewing sarcoma/peripheral primitive neuroectodermal tumor of the vulva. *Hum Pathol*.

[10] Halil S, Kucuk M, Arvas M, Aydin O, Calay ZZ (2011). Peripheral primitive neuroectodermal tumor (PNET) of the vulva: a case report. *Eur J Gynaecol Oncol*.

[11] Kelling K, Noack F, Altgassen C, Kujath P, Bohlmann MK, Hoellen F (2012). Primary metastasized extraskeletal Ewing sarcoma of the vulva: report of a case and review of the literature. *Arch Gynecol Obstet*.

[12] Narayanan G, Rajan V, Puthusseri J, Kattoor J, Soman LV (2014). Primitive neuroectodermal tumor of the vulva in an adolescent girl. *World J Oncol*.

[13] Rekhi B, Qureshi S, Basak R (2010). Primary vaginal Ewing’s sarcoma or primitive neuroectodermal tumor in a 17-year-old woman: a case report. *J Med Case Rep*.

[14] Kakoti LM, Sharma JD, Kataki AC, Barmon D (2017). Primary Ewing sarcoma of vulva: a case report and a review of literature. *Indian J Gynecol Oncol*.

[15] Xu QQ, Xing WW, Chen G (2019). Primitive neuroectodermal tumors of the abdominal wall and vulva in children: report of two cases and review of the literature. *World J Clin Cases*.

[16] McCluggage WG, Sumathi VP, Nucci MR (2007). Ewing family of tumors involving the vulva and vagina: report of a series of four cases. *J Clin Pathol*.

[17] Jiang S, Wang G, Chen J, Dong Y (2018). Comparison of clinical features and outcomes in patients with extraskeletal vs skeletal Ewing sarcoma: an SEER database analysis of 3,178 cases. *Cancer Manag Res*.

[18] Cotterill SJ, Ahrens S, Paulussen M (2000). Prognostic factors in Ewing’s tumor of bone: analysis of 975 patients from the European Intergroup Cooperative Ewing’s Sarcoma Study Group. *J Clin Oncol*.

[19] Che SM, Cao PL, Chen HW, Liu Z, Meng D (2013). Primary Ewing’s sarcoma of vulva: a case report and a review of the literature. *J Obstet Gynaecol Res*.

